# Diallyl Disulfide (DADS) Ameliorates Intestinal *Candida albicans* Infection by Modulating the Gut microbiota and Metabolites and Providing Intestinal Protection in Mice

**DOI:** 10.3389/fcimb.2021.743454

**Published:** 2022-01-07

**Authors:** Wanchao Hu, Liou Huang, Ziyang Zhou, Liping Yin, Jianguo Tang

**Affiliations:** Department of Trauma-Emergency & Critical Care Medicine, Shanghai Fifth People’s Hospital, Fudan University, Shanghai, China

**Keywords:** diallyl disulfide, *Candida albicans*, gut barrier, gut microbiota, gut metabolites

## Abstract

Diallyl disulfide (DADS), a garlic extract also known as allicin, has been reported to have numerous biological activities, including anticancer, antifungal, and inflammation-inhibiting activities, among others. Although many studies have assessed whether DADS can treat *Candida albicans* infection *in vitro*, its *in vivo* function and the underlying mechanism are still not clear. Accumulated evidence has implicated the gut microbiota as an important factor in the colonization and invasion of *C. albicans*. Thus, this study aimed to identify the mechanism by which DADS ameliorates dextran sulfate (DSS)-induced intestinal *C. albicans* infection based on the systematic analysis of the gut microbiota and metabolomics in mice. Here, we determined the body weight, survival, colon length, histological score, and inflammatory cytokine levels in the serum and intestines of experimental mice. Fecal samples were collected for gut microbiota and metabolite analysis by 16S rRNA gene sequencing and LC–MS metabolomics, respectively. DADS significantly alleviated DSS-induced intestinal *C. albicans* infection and altered the gut microbial community structure and metabolic profile in the mice. The abundances of some pathogenic bacteria, such as *Proteobacteria*, *Escherichia–Shigella*, and *Streptococcus*, were notably decreased after treatment with DADS. In contrast, SCFA-producing bacteria, namely, *Ruminiclostridium*, *Oscillibacter*, and *Ruminococcaceae_UCG−013*, greatly increased in number. The perturbance of metabolites in infectious mice was improved by DADS, with increases in secondary bile acids, arachidonic acid, indoles and their derivatives, which were highly related to the multiple differentially altered metabolic pathways, namely, bile secretion, arachidonic acid metabolism, and tryptophan metabolism. This study indicated that DADS could modulate gut microbiota and metabolites and protect the gut barrier to alleviate DSS-induced intestinal *C. albicans* infection in mice. Moreover, this work might also provide novel insight into the treatment of *C. albicans* infection using DADS.

## Introduction

*Candida albicans* is one of the most common commensal fungi in humans but causes millions of disseminated infections each year and can even cause death (Wisplinghoff et al., 2004; Bongomin et al., 2017; Pappas et al., 2018; Toda et al., 2019). As an opportunistic pathogen, *C. albicans* is a normal component of the human gut microbiota. Several studies have shown that the gut community could influence the colonization and invasion of *C. albicans* (Mason et al., 2012; Gutierrez et al., 2020). Moreover, gut *C. albicans* can also induce bacteremia and an imbalance in the gut microbiota (Neville et al., 2015; Hiengrach et al., 2019; Valentine et al., 2019). Therefore, symptomatic *C. albicans* infection is closely related to the interplay of *C. albicans* with other gut bacteria. On the other hand, damage to the intestinal barrier also plays an important role in gut microbiota dysbiosis and fungal disease (Yan et al., 2013; Basmaciyan et al., 2019; Kumamoto et al., 2020). Therefore, it is of great importance to understand the factors affecting fungal colonization in order to prevent disease associated with *C. albicans* infection. However, when we establish intestinal *C. albicans* infectious models, mice will excrete gavaged *C. albicans* and cannot be infected. Thus, we used DSS to induce the gut damage and then gavage *C. albicans* (Hiengrach et al., 2019). In our previous studies, we found that cocultivation of pathogenic *Escherichia coli* with *C. albicans* could reduce the colonization of intestinal cells by *C. albicans* and reduce fungal virulence gene expression (Yang et al., 2016). An increasing number of studies have indicated that the intestinal microbiota, metabolites, and gut barrier could affect the colonization and invasion of *C. albicans*. Previous studies using 16S rRNA sequencing method suggested that intestine-derived *C. albicans* can erode the intestinal mucosa by regulating the intestinal flora. Moreover, compared with that in healthy mice, the relative abundances of *Bacteroides*, *Pseudomonas*, and *Enterococcus* were increased significantly, while the abundance of *Firmicutes* (such as *Lactobacillus*) was decreased, in model mice with intestinal *C. albicans* infection. In addition, *C. albicans* has a synergistic pathogenic effect with *Enterococcus*, which can lead to the destruction of the epithelial barrier by reducing the expression of the intestinal epithelial adhesion protein E-cadherin. Additionally, intestinal injury is a prerequisite for disseminated gut *C. albicans* infection (Koh et al., 2008; Bertolini et al., 2019; Hiengrach et al., 2019; Zhai et al., 2020). In addition, the gut microbiota and metabolites are closely related. Nontargeted high-throughput metabolomics analysis technologies provide an opportunity to explore the changes in metabolites related to microbiota community imbalance during disease development. As a part of the gut microbiome, *C. albicans* could also produce some toxic metabolites, such as adhesin and extracellular proteases (Staniszewska, 2020). Moreover, the impact of *C. albicans* on gut flora will also cause changes in the microbiota metabolites, but there are not many studies in this area. Studies indicated that fungi, as a kind of commensal fungi, could promote the production of indole derivatives, such as, tryptophan and indole-3-aldehyde, which could activate AhR to protect and maintain mucosal integrity during fungal infections or chemical damage and induce anticandidal resistance (Heath-Pagliuso et al., 1998; Wikoff et al., 2009; Zelante et al., 2013; Bessede et al., 2014; Romani et al., 2015). Studies have shown that inflammatory bowel disease (IBD) is related to changes in microorganisms and the metabolic environment in the colon, which participate in signal transduction and immune system regulation and affect the activity of antibiotics (Machiels et al., 2014; Imhann et al., 2018; Franzosa et al., 2019). Some metabolites produced by gut bacteria, such as short-chain fatty acids (SCFAs), indoles, bile acids, and amino acids, can regulate the intestinal epithelium and immune function (Sun et al., 2017; Parada Venegas et al., 2019; Lavelle and Sokol, 2020). However, few researchers have combined studies of the intestinal microbiota with studies of the changes in fecal metabolites during intestinal *C. albicans* infection.

Other interesting studies have indicated that traditional Chinese medicines have a certain effect in regulating gut microbiota. Moreover, diet plays an important role in human health through regulating gut microbiome (Gentile and Weir, 2018; Zmora et al., 2019). Garlic, as a food that is often consumed daily, is also a longstanding commonly used Chinese folk medicine. The effective ingredient in garlic is diallyl disulfide (DADS), also known as allicin, which can exert antifungal, antibacterial, and antitumor effects and ameliorate cardiovascular disease (Yi and Su, 2013; He et al., 2021). The antifungal effect of DADS against *C. albicans* involves the inhibition of biofilm formation by preventing the conversion of yeast to hyphae (Khodavandi et al., 2011). Additionally, DADS can reduce oxidative stress and inflammation and inhibit the cell apoptosis induced by *C. albicans* (Lemar et al., 2005). Moreover, DADS also has important effects on cellular immunity and humoral immunity (Yadegari et al., 2009). However, animal experiments involving the treatment of gut *C. albicans* infection with DADS have not yet been reported, and whether the treatment mechanism involves the gut microbiota and the metabolite intestinal barrier has not been studied. In this study, the mechanism of DADS treatment was explored by establishing mouse models with DSS-induced intestinal *C. albicans* infection, which are more representative of the conditions in the human body (Hiengrach et al., 2019).

## Materials and Methods

### Animals and *C. albicans* Culture

Female C57BL/6 mice aged 8 weeks were purchased and housed in the Animal Center of East China Normal University (Shanghai, China). Mice were fed *ad libitum* and allowed to adapt to the environment (24 ± 2°C, 60 ± 5% relative humidity, 12/12 h dark/light cycle) for one week. All animal experiments were approved by the Experimental Animal Ethical Review Committee, East China Normal University (Shanghai, China). *C. albicans* (strain SC5314) was purchased from the China General Microbiological Culture Collection Center (CGMCC) and then cultivated in yeast extract peptone dextrose (YEPD) liquid medium. Next, a loop was inoculated and streaked on chromogenic medium for the detection of *C. albicans* (CHROMagar Company, France). Then, a single colony was streaked on a YEPD agar plate, incubated for 25 h at 35°C and reidentified by mass spectrometry (Shanghai Fifth People’s Hospital, Fudan University, Department of Laboratory). An inoculum of 1.0 × 10^6^ C*. albicans* cells was prepared in 0.3 ml phosphate buffered saline (PBS, pH 7.4).

### Induction of Intestinal *C. albicans* Infection in a Mouse Model With DSS

**Figure 1:**
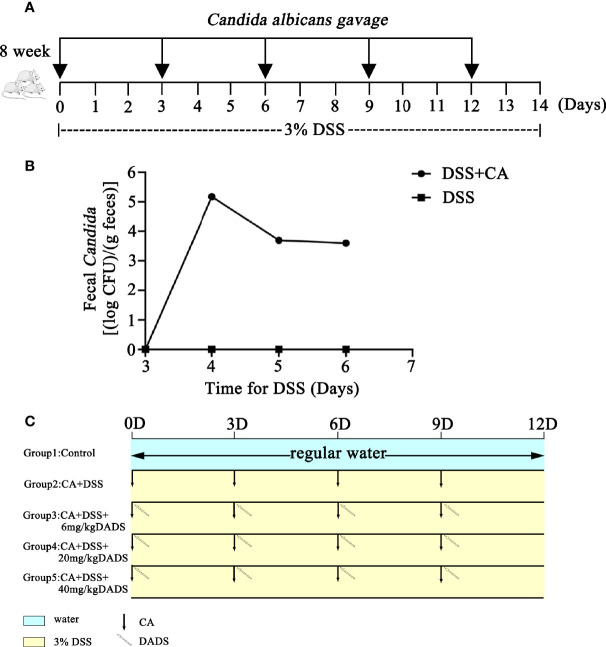
Schematic of the animal experiment design, including *C. albicans* gavage and DADS administration. **(A)** Schematic of *C. albicans* infection in the animal models. **(B)** Fungal burden in feces. **(C)** Schematic diagram of the experiment to compare the function of different concentrations of DADS.

**Figure 2:**
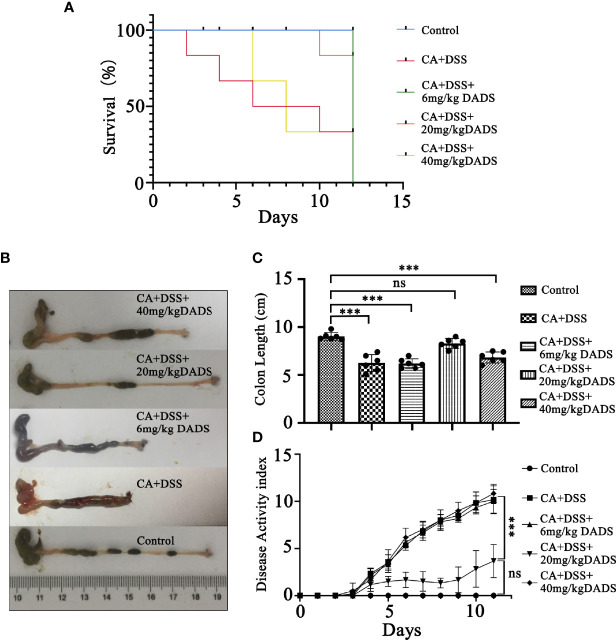
Effects of different concentrations of DADS on mice with *C. albicans* infection. **(A)** Survival study. **(B, C)** Colon length and relevant statistical analysis. **(D)** The disease activity index of animals in each group. Data are presented as the mean ± SEM (n = 6). *P < 0.05, **P < 0.01, ***P < 0.001.

Mouse models of intestinal *C. albicans* infection were constructed as previously published (Hiengrach et al., 2019). Dextran sulfate (DSS; 3% wt/vol, 40 kD, Sigma-Aldrich, USA) was included in the drinking water throughout the entire process to induce colon damage. The PBS yeast suspension was orally administered every 3 days to promote *C. albicans* colonization in the gut (**Figure 1A**). Repeated oral-gastric gavage of *C. albicans* was performed to maintain the fungal load in the gut at a certain level. The fecal *C. albicans* content was evaluated on chromogenic medium (**Figure 1B**). To determine the optimum treatment effect, we tested different concentrations of DADS to treat *C. albicans* infectious mice; 6, 20, and 40 mg/kg were selected based on previous publications (Benavides et al., 2007; Yousuf et al., 2011; Alam et al., 2013; Liang et al., 2015; Motta et al., 2015; Zhang et al., 2019). DADS was diluted in 0.3 ml PBS and administered by oral-gastric gavage after 6 h of treatment with *C. albicans* (**Figure 1C**). A dosage of 20 mg/kg DADS was determined to be suitable for subsequent experimental groups through an evaluation of survival rate, colon length and disease activity index (**Figure 2**). Seventy-five female mice were randomly divided into five groups: control, DSS, CA + DSS, CA + DSS + DADS (20 mg/kg), and DSS + DADS. After fifteen days of treatment, feces were collected from all mice and stored at −80°C. Blood was sampled by retro-orbital puncture, after which the mice were sacrificed by cervical dislocation and their colons and spleens were harvested.

### Histomorphological Analysis

Colon samples were fixed in 4% neutral paraformaldehyde solution, dehydrated, embedded in paraffin, sectioned into 5-μm-thick slices and then stained with hematoxylin and eosin (H&E) for observation. Histological changes were assessed by two blinded experienced pathologists at the same time using a previously described scoring system (Williams et al., 2001; Christophi et al., 2016), and the average score was taken.

### Western Blot Analysis

To analyze the protein expression of colon tissues, western blot analysis was performed according to standard methods. Primary antibodies against Occludin (Proteintech, 13409-1-AP), Claudin-1 (GeneTex, GTX54539), and β-actin (Cell Signaling Technology, CST-3077) were used. The secondary antibodies were obtained from Jackson ImmunoResearch company: anti-mouse IgG (115-035-003) and anti-rabbit IgG (111-035-003).

Then, the protein bands were visualized using an ECL chemiluminescence imaging system and quantified by ImageJ software to calculate the ratios of IntDen (target protein)/IntDen (β-actin).

### Enzyme-Linked Immunosorbent Assays

Mouse blood samples were collected and then centrifuged at 3,000 rpm at 4°C for 10 min for serum collection. Colon tissues were ground in 9× fold homogenization medium and then centrifuged for 10 min at 3,000 rpm and 4°C to collect the supernatants. All serum and tissue supernatants were stored at −80°C for later simultaneous detection. Subsequently, the levels of IL-6 and IFN-γ were measured by murine ELISA kits (88-7064, Thermo Fisher, Austria; EK280/3-01, MuLTI SCIENCE, Shanghai) according to the manufacturer’s instructions.

### Measurement of FITC-Dextran Leakage

As previously described (Watson et al., 2015), FITC-dextran leakage was measured to evaluate gut permeability. Briefly, mice were starved overnight for approximately 8 h and then administered 25 mg/ml FITC-dextran (4 kDa, Sigma-Aldrich, USA) dissolved in PBS. FITC-dextran was gavaged at a dose of 0.6 mg per gram of body weight. Blood samples (400 μl) were collected *via* retro-orbital puncture after 4 h. Then, the supernatants were collected by centrifugation and mixed with an equivalent amount of PBS. Afterward, the fluorescence intensity of diluted serum (100 μl) from each sample was detected using a multimode reader (excitation: 485 nm emission: 528 nm, bandwidth: 20 nm). The quantity of FITC was calculated with a standard curve.

### 16S rRNA Analysis of the Microbial Community

The composition of the gut microbiota and profile of metabolites were assessed as previously described (Hu et al., 2021). Feces were collected from each group of mice. Then, we randomly selected 6 fecal pellets for microbiome and metabolomics analyses. Briefly, DNA was extracted from feces by a Standard DNA Extraction Kit (QIAGEN). Then, the quality and quantity of DNA were confirmed by agarose gel electrophoresis. The V3–V4 regions of the 16S rRNA genes were amplified, and the quality was verified; the amplicon was then purified and amplified again. Sequencing of the V3–V4 gene amplicons was obtained using the Illumina MiSeq platform. The raw data were filtered, and clean tags were removed to obtain valid tags for preparing operational taxonomic units (OTUs), which were classified using Vsearch software (version 2.4.2) (Rognes et al., 2016) with a threshold of 97% sequence similarity. Subsequently, according to the sequence comparison of OTUs, pynast (v0.1) software (Caporaso et al., 2010) was used to construct a phylogeny. The diversity and composition of the intestinal microbiota were determined based on a rarefied OTU table. Alpha diversity indexes of fecal samples were generated from a normalized OTU table at a uniform depth. Beta diversity indexes were generated to determine whether significant differences in gut microbiota existed among different groups based on the Bray–Curtis algorithm and unweighted UniFrac distance and were also determined by principal component analysis (PCA).

### Fecal Metabolome Analysis

LC–MS analysis was performed by OE BioTech (Shanghai, China). Fecal sample preparation and analysis were performed as previously described (Liu et al., 2020). Briefly, fecal pellets (60 mg) were mixed with 500 μl of solvent and then ground, vortexed and centrifuged for 15 min at 13,000 rpm at 4°C. Subsequently, the supernatant was filtered using a 0.22 μm microfilter, and the resulting supernatant was stored at −80°C for LC–MS analysis. The quality control (QC) group was established by pooling equal volumes of supernatant from each sample to determine whether the mass spectrum platform of the system was stable during the whole experiment. The metabolite profiles were analyzed on an AB TripleTOF 6600 mass spectrometer (AB Sciex) combining ESI sources in both positive and negative ion scan modes. TOF parameters were as described previously (Xiong et al., 2019). All reagents used were of high-performance liquid chromatography (HPLC) grade.

The LC–MS data from fecal pellets were processed by Progenesis QI software (Waters Corporation, Milford, USA), and then Progenesis QI Data Processing Software was used to identify the metabolites. The normalized data were visualized by PCA and orthogonal partial least squares-discriminant (OPLS-DA) analysis using the ropls package in R. The ellipses in PCA and OPLS-DA plots were employed to characterize metabolic perturbation among groups in a Hotelling T2 region with a 95% confidence interval threshold.

The variable importance in projection (VIP) was calculated based on the OPLS-DA model to identify significant metabolites with a VIP >1.0 and *P-value <*0.05. The KEGG (http://www.kegg.com/) database was used to explore the related metabolic pathways.

### Statistical Analysis

Statistical analysis was carried out by SPSS 24.0 software (SPSS Inc., Chicago, IL). All data were calculated from no fewer than three replicates and are presented as the mean ± S.E.M. All data were tested for normal distribution before comparisons between groups. If the data were normally distributed, the differences between groups were examined for statistical significance by Student’s t-test (for comparisons between two groups) or one-way ANOVA (for comparisons between multiple groups). Otherwise, nonparametric tests, including the Wilcoxon test, Bray–Curtis distance, Euclidean distance, and UniFrac, were used to analyze the gut microbiota and metabolite data. Survival analysis was performed by log-rank test. The analysis methods used are provided in the figure legends. A *P-*value <0.05 was considered statistically significant.

## Results

### DSS-Induced Intestinal *C. albicans* Infection Alleviated by 20 mg/kg DADS in Mice

We evaluated whether DADS could exert antifungal and anti-inflammatory effects in gut *C. albicans* infectious mouse models (**Figure 1**). The fecal *C. albicans* level was maintained at 10^4^ CFU/g feces after treatment with 3% DSS and repeated gavage with *C. albicans* (**Figures 1A, B**).

**Figure 3:**
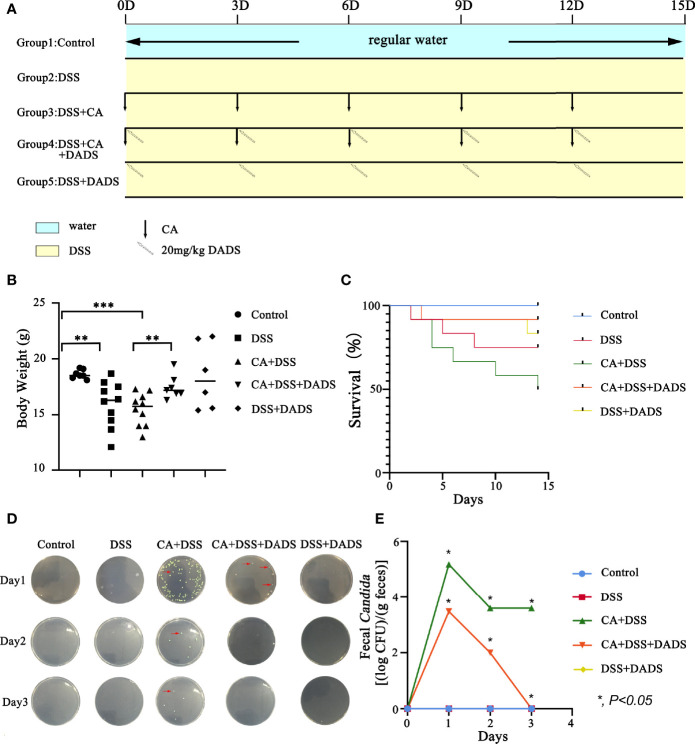
DADS alleviated symptoms in mice with *C. albicans* infection. **(A)** Schematic of *C. albicans* gavage infection and 20 mg/kg DADS administration. **(B)** Changes in body weight. **(C)** Survival study. **(D, E)** Fungal burden in feces.

Infected mice were treated with three concentrations of DADS: 6, 20, and 40 mg/kg (**Figure 1C**). Mice with intestinal *C. albicans* infection exhibited a decreased survival rate and shortened colon, both of which were improved by administration of 6 and 20 mg/kg DADS but not 40 mg/kg DADS (**Figures 2A–C**). Meanwhile, treatment with 20 mg/kg DADS caused a reduction in the disease activity index (DAI), consistent with the findings of weight loss, general condition and fecal occult blood tests (**Figure 2D**). From the observations above, 20 mg/kg was regarded as the optimum concentration of DADS for the following experimental grouping (**Figure 3A**). Mice treated with DSS to induce colon damage were considered the model control group (DSS), and mice treated with DSS + DADS were the experimental group. While administration DSS and *C. albicans* significantly decreased the body weight and survival of mice, DADS treatment alleviated these effects (**Figures 3B, C**). Moreover, DADS treatment caused a marked decrease in the load of fecal *C. albicans* compared with the CA + DSS group (**Figures 3D, E**). These data suggested that it is better to alleviate DSS-induced intestinal *C. albicans* infection in mice with 20 mg/kg DADS treatment, compared with 6 mg/kg. As for 40 mg/kg DADS, there is no improvement.

### DADS Exhibited an Anti-Inflammatory Effect and Protected the Epithelial Barrier of Mice With DSS-Induced Intestinal *C. albicans* Infection

**Figure 4:**
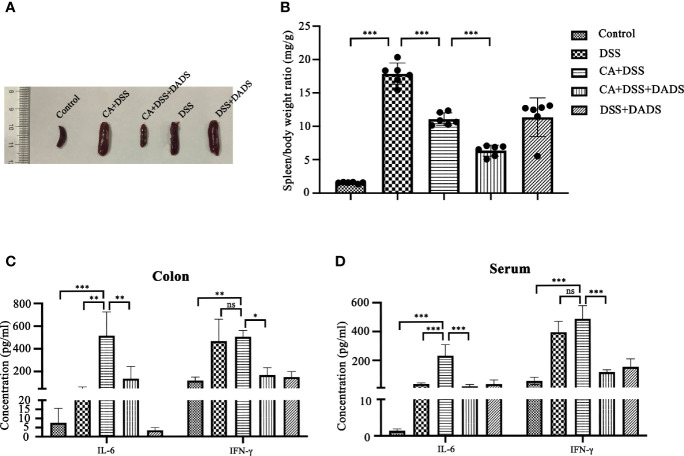
DADS exhibited an anti-inflammatory effect in mice with *C. albicans* infection. **(A, B)** Spleen size and the spleen/body weight ratio. **(C, D)** The concentrations of IL-6 and IFN-γ in the colon and serum.

**Figure 5:**
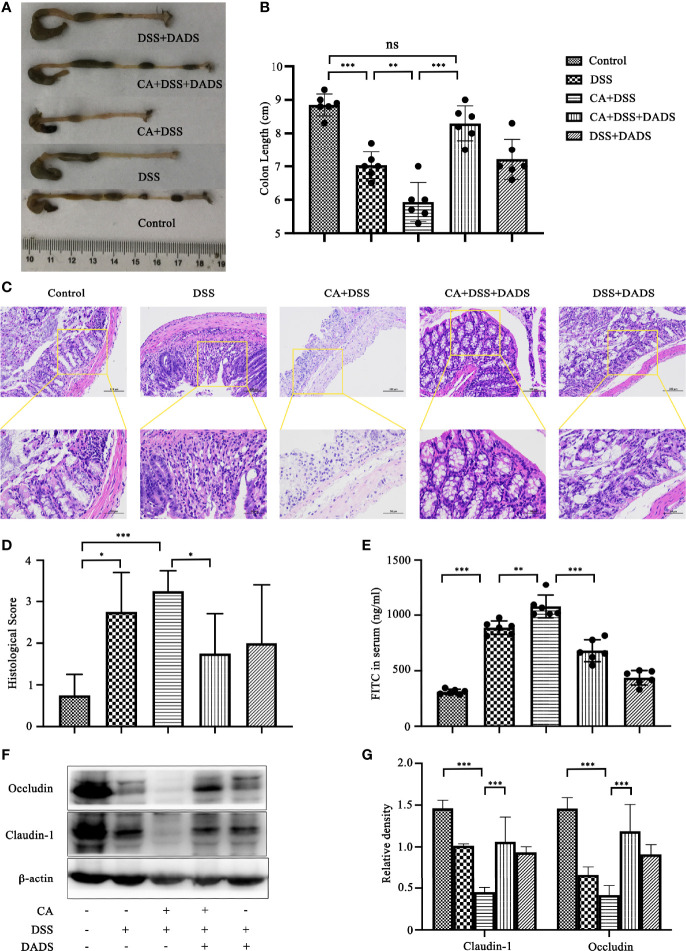
DADS protected the epithelial barrier of mice with *C. albicans* infection. **(A, B)** Colon length and relevant statistical analysis. **(C, D)** H&E staining of colon pathological changes and relevant scores. **(E)** FITC levels in the serum. **(F, G)** The levels of tight junction proteins, including Clauding-1 and Occludin. *P < 0.05, **P < 0.01, and ***P < 0.001.

Spleen size can reflect inflammation in mice. DSS and *C. albicans* increased the spleen/body weight ratio, which was significantly improved by DADS (**Figures 4A, B**). In addition, we employed ELISA to quantify the expression of proinflammatory cytokines, namely, IL-6 and IFN-γ. Notably, DSS and *C. albicans* caused a significant increase in IL-6 and IFN-γ in both the serum and colon, and these changes could be reversed by DADS treatment (**Figures 4C, D**). To investigate the effect of DADS on intestinal inflammation, the length of colons and histological changes were compared among groups. Consistent with the above results, DADS ameliorated the colon shortening observed in mice treated with DSS and *C. albicans* (**Figures 5A, B**). The histological examination results revealed partial loss of the mucosal glands, erosion of the submucosal and muscular layers, and increased neutrophil infiltration in mice with *C. albicans* infection, which was alleviated by DADS (**Figures 5C, D**). Damage to the intestine was also evaluated by quantifying the FITC content in the serum, which indicated that the increased permeability in the DSS and CA + DADS groups could be improved by DADS (**Figure 5E**). Furthermore, the expression levels of the tight junction proteins Claudin-1 and Occludin in the colon were also quantitatively analyzed; these proteins were significantly depleted in intestinal *C. albicans*-infected mice and improved in the CA + DSS + DADS group **(** **Figures 5F, G** **)**. These data demonstrated that DADS treatment could ameliorate the damage to the intestinal barrier caused by DSS and *C. albicans*. 

### DADS Altered the Gut Microbiota Community Composition of Mice With DSS-Induced Intestinal *C. albicans* Infection

**Figure 6:**
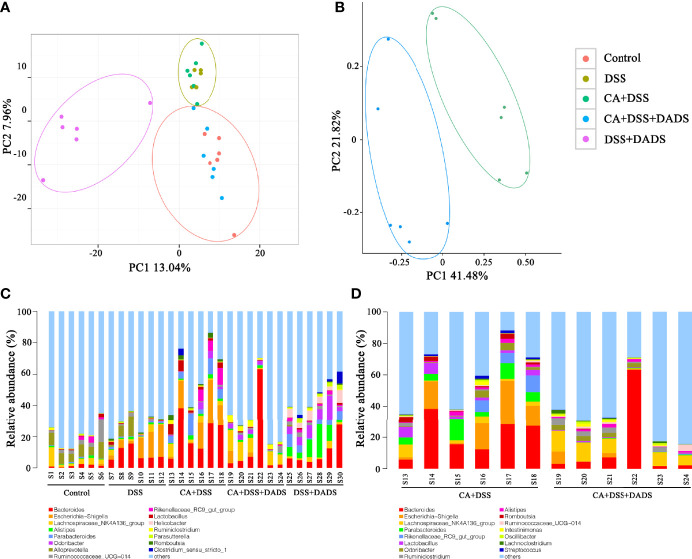
DADS altered the gut microbiota community composition of mice with *C. albicans* infection. **(A, B)** Principal component analysis. **(C, D)** The relative abundances of the top 15 bacteria at the genus level among groups. *P < 0.05, **P < 0.01.

Many studies have indicated that certain gut microbiota could influence the survival and colonization properties of *C. albicans*. During inflammation of the colon and DADS treatment, the gut microbial communities may be altered. Based on this model, 16S rRNA gene sequencing was used to identify key bacteria and relevant metabolic pathways that might be changed among groups. More than 99.4% of the sequence exhibited good coverage values, which indicated adequate sequencing depth for all groups. All other alpha diversity values (OTUs, observed species, Chao1, and Shannon index) were lower in mice after treatment with DSS and/or *C. albicans* than in the control group and were not improved by DADS (**Table 1** **)**. PCA showed that *C. albicans*, DSS, and DADS treatment induced changes in the intestinal microbiota composition. Moreover, the fecal samples from the DSS and CA + DSS groups were clustered together with high similarity, but in DADS-treated mice, samples from the CA + DSS + DADS group were clustered with the control samples **(** **Figure 6A** **)**. PCA also separated the CA + DSS and CA + DSS + DADS groups, which indicated that these gut microbial communities were significantly different **(** **Figure 6B** **)**. As shown in **Figures 3C, D**, we determined the relative abundances of the top 15 bacteria at the genus level among the groups. The genera *Bacteroides*, *Escherichia–Shigella*, *Lachnospiraceae_ NK4A136_group*, *Parabacteroides*, and others exhibited high relative abundance in all groups. Compared with *C. albicans*-infected mice, the DADS treatment group had decreased relative abundances of *Escherichia–Shigella* and *Parabacteroides.*

**Table 1:** The alpha diversity in each group of mice.

Items	Control	DSS	CA + DSS	CA + DSS + DADS	DSS + DADS
OTUs	598.83 ± 27.72	462.67 ± 41.47	484.5 ± 63.4	470.83 ± 33.09	572.57 ± 72.04
Chao1	771.81 ± 56.89	677.52 ± 42.86	687.99 ± 53.67	642.59 ± 43.3	808.85 ± 62.42
Goods_coverage	0.99502 ± 0.00066	0.9948 ± 0.00027	0.99486 ± 0.00028	0.99556 ± 0.00042	0.99445 ± 0.00034
Observed_species	598.8 ± 29.95	459.1 ± 38.36	478.87 ± 58.82	468.18 ± 29.91	593.93 ± 58.77
Shannon	5.43 ± 0.49	3.53 ± 0.6	4.8 ± 0.54	5.07 ± 0.96	5.9 ± 0.3
Simpson	0.93 ± 0.03	0.75 ± 0.12	0.92 ± 0.02	0.9 ± 0.07	0.96 ± 0.01

**Figure 7:**
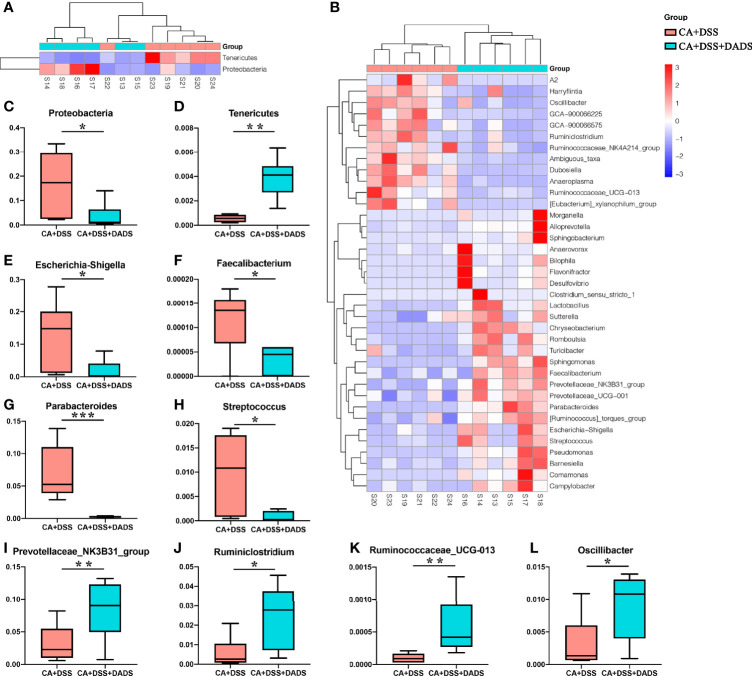
Heatmaps of key bacteria at the **(A)** phylum and **(B)** genus levels. The relative abundances of **(C)** *Proteobacteria*, **(D)** *Tenericutes*, **(E)** *Escherichia–Shigella*, **(F)** *Faecalibacterium*, **(G)** *Parabacteroides*, **(H)** *Streptococcus*, **(I)** *Prevotellaceae_NK3B31_group*, **(J)** *Ruminiclostridium*, **(K)** *Ruminococcaceae_UCG-013*, and **(L)** *Oscillibacter* in the two groups.

The key bacteria were visualized by a heatmap and analyzed by the Wilcoxon rank-sum test (**Figures 7A, B**). At the phylum level, DADS treatment was associated with a decreased abundance of Proteobacteria (P = 0.019, **Figure 7C**) and increase abundance of Tenericutes (P = 0.002, **Figure 7D**) compared to the intestinal C. albicans infectious group (P < 0.05) (**Figure 7A**). As shown in **Figure 7B**, at the genus level, the abundances of *Escherichia–Shigella* (*P* = 0.014, **Figure 7E**), *Faecalibacterium* (*P* = 0.027, **Figure 7F**)*, Parabacteroides* (*P* = 0.001, **Figure 7G**), and *Streptococcus* (*P* = 0.023, **Figure 7H**) were significantly decreased, and the abundances of *Prevotellaceae_NK3B31_group* (*P* = 0.002, **Figure 7I**), *Ruminiclostridium* (*P* = 0.023, **Figure 7J**), *Ruminococcaceae_UCG-013* (*P* = 0.002, **Figure 7K**), and *Oscillibacter* (*P* = 0.038, **Figure 7L**) were enriched in DADS-treated mice (**Table 2**).

**Table 2:** Changes of gut microbiota at different levels among the two groups.

Phylum/class	Family/genus	Relative contribution^a^		Fold change^b^	p-value^c^
		CA+DSS	CA+DSS+DADS		
Firmicutes	–	23.20%	31.14%	1.342660362	0.392
	Faecalibacterium	0.01%	0.0035%	0.304347826	0.027
	Streptococcus	0.99%	0.08%	0.083713851	0.023
	Ruminiclostridium	0.58%	2.46%	4.263660017	0.023
	Ruminococcaceae_UCG-013	0.01%	0.06%	5.75	0.002
	Oscillibacter	0.32%	0.90%	282.03%	0.038
Proteobacteria	–	16.85%	3.46%	0.205146491	0.019
	Escherichia–Shigella	1.82%	12.79%	7.018131868	0.014
Tenericutes	–	0.06%	0.39%	6.89380531	0.002
Bacteroidetes	–	59.62%	64.62%	1.083814932	0.694
	Parabacteroides	6.93%	0.26%	0.03698086	0.001
	Prevotellaceae_NK3B31_group	0.01%	0.0015%	0.136363636	0.002

^a^Relative contribution, ^b^Fold change, ^c^p-value.

### DADS Improved the Fecal Metabolite Profiles of Mice With DSS-Induced Intestinal *C. albicans* Infection

**Figure 8:**
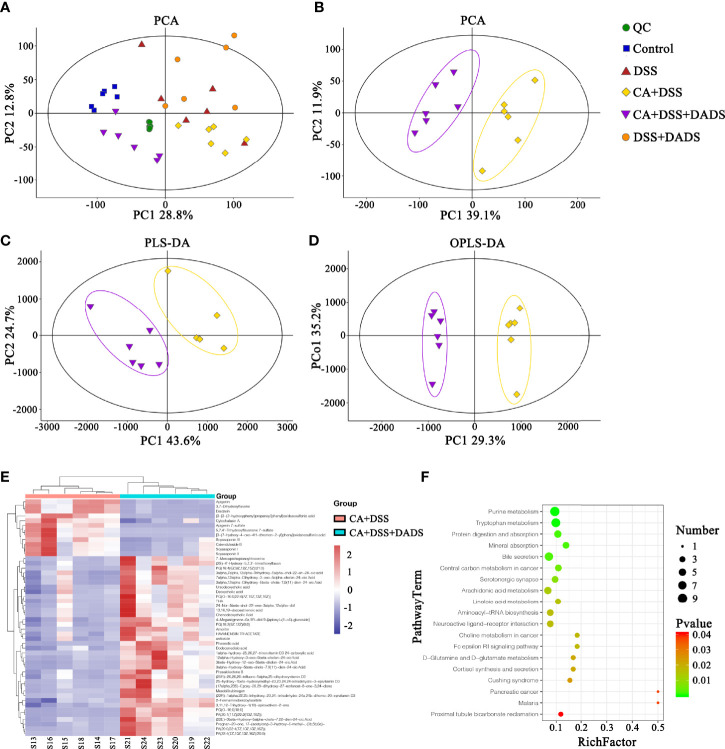
Effect of DADS on fecal metabolites in mice with *C. albicans* infection. The **(A, B)** PCA, **(C)** PLS-DA, and **(D)** OPLS-DA models in different groups. **(E)** Heatmaps of differentially altered metabolites between the two groups. **(F)** Differential metabolic pathways visualized in bubble plots (*P <* 0.05); the bubble size represents the number of metabolites, n = 6.

Metabolic changes are closely related to alterations of the gut microbiota and are also considered a crucial hallmark of intestinal inflammation (Lanis et al., 2017). Thus, we performed LC–MS analysis to detect differentially expressed metabolites and relevant key metabolic pathways among groups. A total of 8,689 metabolites were identified in 30 fecal samples among the five groups. The PCA scatter plots showed clustered QC samples, which indicated the high quality of metabolomics analysis **(** **Figure 8A** **)**. We further identified the differences in the metabolic profile between the CA + DSS and 20 mg/kg DADS treatment groups using two-dimensional PCA, PLS-DA, and OPLS-DA analysis **(** **Figures 8B–D** **)**. Next, to identify key metabolites, we visualized the top 50 metabolites on a heatmap and identified nineteen metabolic pathways that were significantly differentially expressed between the *C. albicans*-infected mice and DADS-treated mice **(** *P <*0.05, **Figures 8E, F** **)**. Additionally, we identified seventeen important metabolites for further analysis between the two groups (**Table 3**). All differentially expressed metabolites related to tryptophan metabolism were upregulated in the DADS group, including 4-(2-aminophenyl)-2,4-dioxobutanoic acid, kynurenic acid, N-acetylisatin, 5-hydroxyindoleacetic acid, quinoline-4,8-diol, 4-(2-amino-3-hydroxyphenyl)-2,4-dioxobutanoic acid, 3-methyldioxyindole, and 2-formaminobenzoylacetate. The levels of some metabolites, such as PGB2, PGD2, TXA2, and lipoxin A4, were increased in DADS-treated mice compared with *C. albicans*-infected mice. Moreover, some metabolites of bile secretion pathways were at a higher level in DADS-treated mice, including deoxycholic acid, lithocholic acid, chenodeoxycholic acid, and cholic acid.

**Table 3:** Differential fecal metabolites and relevant pathways between two groups.

Number	Metabolites	KEGG ID	VIP^a^	Relative concentration		Model VS Control		Metabolic pathway	Classification
				CA + DSS	CA + DSS + DADS	FC^b^	P-value^c^		
1	4-(2-Aminophenyl)-2,4-dioxobutanoic acid	C01252	5.11	3006.75 ± 774.77	5,814.92 ± 753.11	1.93	0.0001	Tryptophan metabolism	Organooxygen compounds
2	Kynurenic acid	C01717	3.09	869.16 ± 341.31	1,893.54 ± 202.69	2.18	0.0001	Tryptophan metabolism	Quinolines and derivatives
3	N-Acetylisatin	C02172	2.27	764.47 ± 178.48	1,329.03 ± 149.9	1.74	0.0001	Tryptophan metabolism	Unclassified
4	5-Hydroxyindoleacetic acid	C05635	3.19	842.47 ± 275.36	1,967.16 ± 388.89	2.33	0.0002	Tryptophan metabolism|Serotonergic synapse	Indoles and derivatives
5	Quinoline-4,8-diol	C05637	4.42	3028.48 ± 694.56	5,182.43 ± 587.04	1.71	0.0002	Tryptophan metabolism	Quinolines and derivatives
6	4-(2-Amino-3-hydroxyphenyl)-2,4-dioxobutanoic acid	C05645	1.13	19.13 ± 5.42	167.84 ± 91.38	8.78	0.0026	Tryptophan metabolism	Organooxygen compounds
7	3-Methyldioxyindole	C05834	1.16	209.32 ± 84.2	389.78 ± 118.85	1.86	0.0126	Tryptophan metabolism	Indoles and derivatives
8	2-Formaminobenzoylacetate	C05835	8.52	10312.75 ± 2153.38	1,8075.57 ± 1,919.96	1.75	0.0001	Tryptophan metabolism	Unclassified
9	TXA2	C02198	1.22	30.74 ± 9.6	178.88 ± 37.47	5.82	0.0000	Serotonergic synapse	Fatty Acyls
10	PGB2	C05954	1.21	65.36 ± 29.6	224.88 ± 70.48	3.44	0.0005	Serotonergic synapse	Fatty Acyls
11	PGD2	C00696	1.12	6.21 ± 4.59	203.97 ± 210.74	32.87	0.0444	Serotonergic synapse	Fatty Acyls
12	Lipoxin A4	C06314	1.75	521.63 ± 208.05	891.74 ± 97.67	1.71	0.0028	Serotonergic synapse	Fatty Acyls
13	Deoxycholic acid	C04483	6.13	5,889.56 ± 4,018.49	1,1702.38 ± 4,298.96	1.99	0.0361	Bile secretion	Bile acids and derivatives
14	Lithocholic acid	C03990	3.09	269 ± 119.43	1,650.66 ± 1,261.03	6.14	0.0234	Bile secretion	Bile acids and derivatives
15	Lithocholate 3-O-glucuronide	C03033	1.38	150.05 ± 92.62	426.1 ± 200.56	2.84	0.0120	Bile secretion|Pentose and glucuronate interconversions	Steroidal glycosides
16	Chenodeoxycholic Acid	C02528	7.61	2,870.1 ± 3,115	1,1744.1 ± 8,335.01	4.09	0.0347	Bile secretion|Primary bile acid biosynthesis	Bile acids and derivatives
17	Cholic acid	C00695	3.65	1,932.38 ± 998.97	3,691.59 ± 1,072.31	1.91	0.0148	Bile secretion|Primary bile acid biosynthesis	Bile acids and derivatives

^a^Relative contribution, ^b^Fold change, ^c^p-value.

## Discussion

Colonization and invasion of intestinal *C. albicans* are usually the prerequisites for disseminated *C. albicans* infection. To date, although many studies have reported that the gut microbiota, metabolites, and intestinal *C. albicans* could interact with each other, comprehensive research has been lacking, as the specific changes in bacteria and metabolites and regulatory mechanisms vary among animal models of intestinal *C. albicans* infection due to differences in intervention measures and other factors. This study mainly explored the effects of DADS, commonly known as allicin, on the gut microbiota, metabolites, and intestinal barrier of DSS-induced mice infected with enterogenic *C. albicans*. The results indicated that DADS could reduce the intestinal destruction caused by DSS and enterogenic *C. albicans* by increasing the expression of intestinal tight junction proteins, reducing intestinal inflammation (reflected by improvements in survival rate, weight change, colon length change, DAI score, and H&E scores) and reducing the expression of inflammatory factors in the serum and colon. In addition, DADS improved the gut microbiota and intestinal metabolite profiles of mice infected with intestinal *C. albicans* and increased the expression of beneficial bacteria and the expression of related bile acids and amino acids.

With regard to intestinal mucosal damage, *C. albicans* colonization of the gut could break through the intestinal barrier to cause further gut tissue damage, disseminated *C. albicans* infection, and even the death of the host. While it is well recognized that *C. albicans* can induce inflammatory bowel dystrophy, the content of *C. albicans* in the intestines of mice is not as high as that in human intestines, and *C. albicans* cannot colonize the intestines of mice by oral-gastric gavage alone (Kumamoto, 2011; Koh, 2013; Hoarau et al., 2016). Therefore, the oral administration of *C. albicans* in the DSS-induced colitis mouse model may be a better model of the condition in humans. Consistent with previous reports, we observed significant weight loss, decreased survival, increased DAI scores, and greater colonization of *C. albicans* in mice with DSS-induced intestinal *C. albicans* infection (Medrano-Díaz et al., 2018; Hirayama et al., 2020). However, all of the above indicators were significantly improved by 20 mg/kg DADS intervention compared with the control treatment, which proved that DADS is effective in the treatment of intestinal *C. albicans* infection. Meanwhile, the intestinal barrier is one of the target organs that is destroyed by intestinal *C. albicans* and plays an important role in preventing disseminated *C. albicans* disease. We quantified the extent of intestinal destruction by testing the concentration of FITC-dextran in the serum in our experimental groups. FITC-dextran is a fluorescent molecule that can cross a damaged intestinal barrier and be transepithelially transported into the blood. Therefore, the higher the content of fluorescent substances in the blood is, the more serious the destruction of the intestinal tract (Gupta and Nebreda, 2014). The most serious intestinal damage was observed in the CA + DSS group, which showed a significant difference from the DSS group. Moreover, these results indicated that *C. albicans* could aggravate intestinal destruction induced by DSS. In the CA + DSS + DADS group, we observed a significantly decreased concentration of FITC-dextran, which showed that DADS ameliorated the effect of *C. albicans* infection on intestinal permeability. We further compared the intestinal barriers of each group based on intestinal pathology and the expression of intestinal tight junction proteins. In terms of intestinal pathology, colon length can be shortened by DSS-induced intestinal inflammation (Jawhara et al., 2012). We found that the CA + DSS group had a shorter intestinal length and higher intestinal H&E pathological score than the DSS group, indicating that *C. albicans* further increased intestinal inflammation and destruction of the intestinal mucosa. In addition, the expression of intestinal tight junction proteins can reflect the integrity of the intestinal mucosa at the protein level (Buckley and Turner, 2018; Shao et al., 2018). Claudin-1 and Occludin were hardly expressed in the CA + DSS group and were expressed at somewhat higher levels in the DSS group. However, in the DADS group, the expression of tight junction proteins was increased compared with that in the CA + DSS or DSS group, which indicated that DADS could increase the expression of colonic tight junction proteins and repair the damage to the intestinal mucosa. We also explored the condition of inflammation among groups by comparing the spleen index and the levels of IL-6 and IFN-γ in the serum, spleen and colon. We found that the spleen index and the expression of IL-6 and IFN-γ was decreased in the DADS group compared with the DSS and CA + DSS groups, but there was no significant difference among the groups in the expression of inflammatory factors in the spleen, which might be related to differences in expression in various tissues or depletion of these factors in the spleen. Compared with the DSS group, the CA + DSS group expressed more serious intestinal damage and inflammation, but DADS treatment alleviated gut damage and inflammation symptoms, which indicated that DADS could repair the intestinal damage caused by *C. albicans* by protecting gut tissues, increasing the expression of tight junction proteins, and reducing inflammation.

Nowadays, nosocomial bloodstream infections caused by *C. albicans* rank third (Wisplinghoff et al., 2004). Moreover, the *C. albicans* in the blood mainly comes from the intestine (Kullberg and Arendrup, 2015). So the destruction of the intestines is conducive to the colonization, invasion, and infection of *C. albicans*. DADS, as a classic garlic active substance, its antifungal and digestive system protection effects have been confirmed, which is consistent with our above research (Shang et al., 2019). Few people study the association between gut protection and antifungal effect. The intestinal protective effect of DADS may reduce the colonization of *C. albicans*, thereby inhibiting the bloodstream entry of *C. albicans* from the intestine. Secondly, the antifungal effect of DADS reduces the destruction of *C. albican* to the intestine, which may also improve the intestinal barrier. But the detailed interaction mechanism needs further study. In addition, the role of DADS on gut microbiota and metabolites is also important for intestinal protection and anti-fungal.

The community of gut microbiota in the CA + DSS group was also significantly different from that in the healthy control group and the DSS group. Interestingly, similar to what was found in previous research, the abundance of *Bacteroides*, *Bacteroidaceae*, *Proteobacteria*, *Escherichia–Shigella*, *Streptococcus*, and other pathogenic bacteria was increased in mice with DSS-induced intestinal *C. albicans* infection. It is possible that the increased mortality in the CA + DSS group was related to bacteremia caused by pathogenic bacteria that selectively break through the intestinal barrier (Hiengrach et al., 2019). Bacteremia may also cause high levels of inflammation in the serum and intestines and increased levels of inflammatory factors (IL-6, IFN-γ) (Michielan and D’incà, 2015). *Proteobacteria* are typically highly abundant in certain intestinal and extraintestinal diseases with inflammatory manifestations, so it is also considered to be a possible microbial feature of these diseases (Rizzatti et al., 2017). In this study, we found that the abundances of *Proteobacteria* (at the phylum level) and *Escherichia–Shigella* (at the genus level) were increased in the CA + DSS group. Studies have shown that *Proteobacteria* could be a biomarker indicating the instability of the gut microbiota, which can invade intestinal epithelial cells and aggravate intestinal inflammation by releasing endotoxins and lipopolysaccharide (LPS) (Boudeau et al., 1999; Lu and Walker, 2001; Belotserkovsky and Sansonetti, 2018). Consistent with previous experiments using similar mouse models with oral-gastric gavage of *C. albicans*, the CA + DSS group exhibited significant enrichment of *Bacteroides*, which is usually a commensal bacterium in the host intestine but may become a pathogen under certain conditions. Previous studies have reported that *Bacteroides* can produce LPS and a variety of enzymes, thereby enhancing the adhesion of bacteria to the intestinal tissues of the host and protecting them from immune attack, ultimately leading to the destruction of the intestinal epithelium (Wexler, 2007; Sears, 2009). In addition, *Bacteroides* can produce enterotoxin to cleave tight junction proteins in the intestine, leading to cytoskeletal rearrangement and loss of tight junctions in the intestinal epithelial cells, which may explain the decreased expression of Claudin-1 and Occludin (Wu et al., 1998). In the CA + DSS + DADS group, we observed an increased abundance of *Ruminococcaceae_UCG−013*, *Ruminococcaceae_NK4A214_group*, *Ruminiclostridium*, and *Oscillibacter*, which are usually not abundant in patients with ulcerative colitis. These results might indicate that DADS can increase the abundance of beneficial bacteria such as *Ruminococcus* to reduce intestinal inflammation (Hyams et al., 2019). In addition, *Ruminiclostridium* has been reported to be increased in healthy mice and to produce butyric acid, which can nourish and protect the intestinal epithelium (Machiels et al., 2014). Thus, these findings indicated that DADS treatment might mitigate intestinal *C. albicans* infection by improving the disordered gut microbiota.

Meanwhile, the intestinal metabolite homeostasis was also altered among the groups. Gut metabolites are closely related to some intestinal and extraintestinal diseases, such as inflammatory bowel disease, irritable bowel syndrome, depression, and autism (Duboc et al., 2012; Bjerrum et al., 2015; Sharon et al., 2019; Keshteli et al., 2019). Moreover, some gut metabolites could protect or destroy the gut barrier (Li et al., 2019; Parada Venegas et al., 2019). In this study, the intestinal metabolite profiles were significantly different between the CA + DSS and CA + DSS + DADS groups. In addition, through KEGG data analysis, we found 19 different metabolic pathways. In terms of the bile acid secretion metabolism, the content of bile acids, such as deoxycholic acid, chenodeoxycholic acid, ursodeoxycholic acid, lithocholic acid, and cholic acid, was significantly higher in the CA + DSS + DADS group than in the CA + DSS group, similar to what has been found in patients with inflammatory bowel disease (Jansson et al., 2009; Duboc et al., 2013). Deoxycholic acid could inhibit the secretion of IL-1β and IL-8 by intestinal epithelial Caco-2 cells in a dose-dependent manner (Yao et al., 2019; Lavelle and Sokol, 2020). The metabolites involved in arachidonic acid metabolism, such as TXA2, PGD2, and PGB2, were significantly increased in the CA + DSS + DADS group. Studies have shown that arachidonic acid metabolites could increase the sensitivity of *C. albicans* to fluconazole (Kuloyo et al., 2020). All metabolites involved in the tryptophan metabolic pathway were enriched in the CA + DSS + DADS group and were significantly higher than in the CA + DSS group. Research has proven that tryptophan metabolism plays a key role in regulating the immune response of the body against *C. albicans.* Tryptophan could activate the microbial-dependent AhR/IL-22 axis to inhibit fungal growth and infection on the mucosal surface and thus prevent abnormal immune stimulation by *C. albicans* (Romani et al., 2008). In addition, the interaction between tryptophan metabolites and *C. albicans* may reduce the toxicity of the fungus (Mayr et al., 2005). Activation of the tryptophan metabolic pathway can produce bioactive molecules to maintain the homeostasis of the intestinal mucosa, which makes tryptophan metabolites the main players in intestinal health (Renga et al., 2019). Therefore, these findings indicated that DADS may inhibit the invasion of intestinal *C. albicans* and ameliorate intestinal barrier damage by activating specific metabolic pathways and increasing the levels of related metabolites.

## Conclusion

In this study, we established DSS-induced intestinal *C. albicans* mouse models to better simulate human intestinal fungal infections. We found that *C. albicans* could enhance DSS colitis severity and that DADS treatment could improve intestinal dysbiosis (altered gut microbiota and metabolites), gut permeability and systemic inflammatory responses. Insight into the effects of DADS treatment may provide novel treatment strategies for intestinal *C. albicans* infection.

## Data Availability Statement

The original contributions presented in the study are publicly available. This data can be found in NCBI under accession number PRJNA778766, and at MetaboLights under accession number MTBLS3750.

## Ethics Statement

The animal study was reviewed and approved by the Experimental Animal Ethical Review Committee, East China Normal University.

## Author Contributions

WH: Conceptualization, Methodology, Investigation, Formal analysis, Writing—Original Draft, Visualization. LH: Conceptualization, Software, Resources. ZZ: Data Curation, Formal analysis. LY: Investigation, data collection, Fecal samples collection, Resources. JT: Conceptualization, Methodology, Writing—Review & Editing, Supervision. All authors contributed to the article and approved the submitted version.

## Funding

This study was funded by the Fifth People’s Hospital of Shanghai, Fudan University (grant number 2018WYZD03, 2020WYZDZK06) and the Shanghai Science and Technology Commission (grant number 18411950600).

## Conflict of Interest

The authors declare that the research was conducted in the absence of any commercial or financial relationships that could be construed as a potential conflict of interest.

## Publisher’s Note

All claims expressed in this article are solely those of the authors and do not necessarily represent those of their affiliated organizations, or those of the publisher, the editors and the reviewers. Any product that may be evaluated in this article, or claim that may be made by its manufacturer, is not guaranteed or endorsed by the publisher.
